# Home Range Utilisation and Territorial Behaviour of Lions (*Panthera leo*) on Karongwe Game Reserve, South Africa

**DOI:** 10.1371/journal.pone.0003998

**Published:** 2008-12-22

**Authors:** Monika B. Lehmann, Paul J. Funston, Cailey R. Owen, Rob Slotow

**Affiliations:** 1 Department of Nature Conservation, Tshwane University of Technology, Pretoria, South Africa; 2 K.e.r.i. Research, Ecological Institute of Research, Schagen, South Africa; 3 School of Biological and Conservation Sciences, University of KwaZulu-Natal, Westville Campus, University of KwaZulu-Natal, Durban, South Africa; University of Exeter, Cornwall Campus, United Kingdom

## Abstract

Interventionist conservation management of territorial large carnivores has increased in recent years, especially in South Africa. Understanding of spatial ecology is an important component of predator conservation and management. Spatial patterns are influenced by many, often interacting, factors making elucidation of key drivers difficult. We had the opportunity to study a simplified system, a single pride of lions (*Panthera leo*) after reintroduction onto the 85 km^2^ Karongwe Game Reserve, from 1999–2005, using radio-telemetry. In 2002 one male was removed from the paired coalition which had been present for the first three years. A second pride and male were in a fenced reserve adjacent of them to the east. This made it possible to separate social and resource factors in both a coalition and single male scenario, and the driving factors these seem to have on spatial ecology. Male ranging behaviour was not affected by coalition size, being driven more by resource rather than social factors. The females responded to the lions on the adjacent reserve by avoiding the area closest to them, therefore females may be more driven by social factors. Home range size and the resource response to water are important factors to consider when reintroducing lions to a small reserve, and it is hoped that these findings lead to other similar studies which will contribute to sound decisions regarding the management of lions on small reserves.

## Introduction

Interventionist conservation management of territorial large carnivores has increased in recent years, especially in South Africa, where farmland has been rehabilitated to game reserves and many species have been reintroduced [1]–[3]. At least 37 reserves have reintroduced lions (*Panthera leo*) primarily for ecotourism, but also for ecological processes [3]–[5]. Understanding of spatial ecology is an important component of these two management objectives, both in planning prior to reintroduction, and in subsequent population management to ensure that the population introduced is not above carrying capacity and is representative of a natural population in terms of size and structure. Because these reserves manipulate both the resource (e.g. water provision and harvesting of prey species) and social environment (selective removals or supplementation of lion), separating the different competing drivers of spatial ecology is important in order to make the correct management decisions in such small reserves.

The home range of a carnivore is generally as large as is necessary but as small as possible to satisfy energetic needs [6], [7]. Upper limits are determined by energy expenditure during territorial defence [8] while lower limits are governed by food availability [9]. Adult male lions maintain a territory largely contiguous with that of their home range and discourage rivals from entering these by patrolling, scent-marking and roaring [8]–[10]. Territorial males can protect their cubs from infanticide [11] in two ways: either directly by accompanying the pride and chasing out rival males [10], [12], or indirectly by maintaining the security of a territory [10].

Male lions show territorial behaviour by roaring and scent-marking while patrolling. Territorial displays are expensive because they separate the males from their females, increasing the risk of infanticide by invading males [13], [14]. Furthermore, roaring highlights the location of the males for intruding coalitions [15], [16], and is also energetically expensive, both in terms of the distances covered [8], and the energetic cost of roaring [17].

The two factors, advertisement and resources may influence territory size, shape, and usage in different ways [18], [19]. It is extremely difficult to separate out these two potentially confounding factors in natural circumstances. However, we had the opportunity to test their relative influences in an artificial situation where a single pride male and pair of females existed in a fenced reserve, and a second pride and male were in a fenced reserve adjacent of them. The fences were electrified and effectively lion-proof. Although from a small sample set, we were therefore able to separate our predictions of responses to social influences and resources, and assess their relative input to lion behavioural decisions and subsequent costs.

We predicted that lions would respond towards resources such as prey, water and cover in all directions, but would respond to social influences only in the direction of the adjacent pride. We measured male and female range use, as well as male scent-marking and roaring in different parts of their range. We assess the relative influence that the social factors impose on the resource factors. We had the further advantage of an experimental manipulation of the system, whereby the social system was manipulated by the removal of one of the coalition males while holding resources constant.

## Methods

### Study area

**Figure 1:**
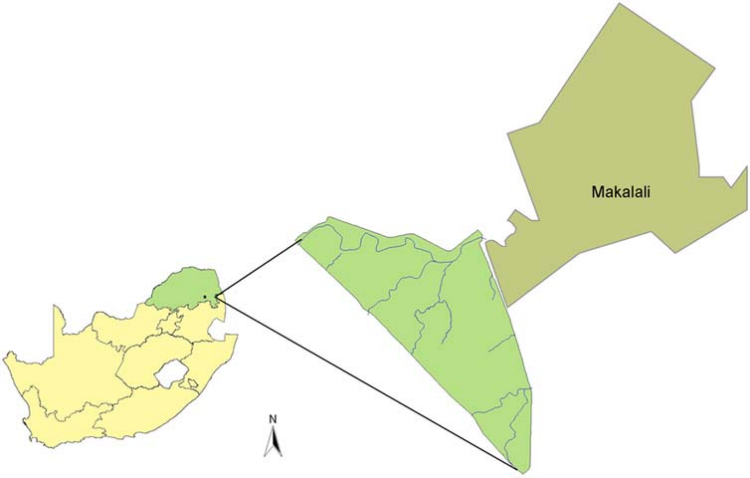
Location of Karongwe Game Reserve and the neighbouring Makalali Game Reserve in Limpopo Province, South Africa.

Fieldwork was conducted on the 85 km^2^ Karongwe Game Reserve (24°13′S and 30°36′E), located in the Limpopo Province, South Africa (Fig. 1). Altitude here varies from 489 m to 520 m above sea level. The reserve falls within the Savanna Biome [20] and lies within the Mixed Lowveld Bushveld [21]. The Greater Makalali Conservancy borders Karongwe on its eastern boundary and is the only other reserve in the area that supports lions, which were introduced to Makalali in 1994 (Fig. 1). Both reserves are fenced with lion-proof electric fences, and there is a road (15 m wide area) separating the two adjacent fences.

As the reserve's main function is eco-tourism a large number of species are present. Apart from lions, other large carnivores include leopards (*Panthera pardus*), cheetahs (*Acinonyx jubatus*), wild dogs (*Lycaon pictus*) and spotted hyeanas (*Crocuta crocuta*), and there are twelve ungulate species as potential prey.

### Field data collection

The study was conducted over a six year period from October 1999 to October 2005, totalling 2192 field days. During that time the lion population varied from four to a maximum of 11 individuals, with an average of eight. After three years, one of the males was removed from the coalition as they were considered to be removing too much prey [22].

A member of each subgroup was located twice daily where possible for the duration of the study (92.3%, *n* = 2024 days), using the standard method for radio telemetry tracking [23]. Most observations took place between 5:00–10:30 (48.4%, *n* = 1809) and 15:30–20:30 (47.7%, *n* = 1782), with some observations at night (3.9%, *n* = 147). The nocturnal observations only covered the period 2004 to 2005 and took place from 22h00 to 02h00. A specific nocturnal study was undertaken to focus on recording territorial behaviour by the pride male from February 2005 to June 2005 (n = 48 nights, 12 sessions of four consecutive nights). Three shift times were chosen: 17:00–23:00, 21:00–04:00, and 23:00–06:00 to incorporate dusk, the middle of the night, and dawn. One session for each shift time in each of the four moon phases was completed. Four nights were spent following the lions continuously from 17:00–06:00.

After locating the focal animal, the following data were recorded: date, time, location, GPS co-ordinate, daily belly score, and general behaviour. The male was followed by vehicle at a distance of 15–30 m. Lions were viewed using a spotlight with a red filter. Any territorial behaviour activities were recorded and georeferenced. All movement and most territorial data were unfortunately only collected after the removal of one male. Therefore no direct comparisons between the one male and a two male coalition's behaviour were possible. We realise that the data set is small (*n* = 1 pride), but we feel that the study nevertheless provides value as more and more managers are stocking such small reserves in a similar manner and can benefit from the experiences observed on Karongwe.

### Data analysis

The data were imported into Arcview 3.2 for home range analyses and the delineation of animal movement paths using the extension Animal Movement [24]. Home ranges were delineated using 95% kernel home ranges for point distributions, and 50% kernels to delineate core areas [25]–[28]. For purposes of this study areas that were defined by the 95% kernel that fell outside the reserve boundary were clipped as these could not contribute to the home range.

Social versus resource drivers of territorial behaviour were contrasted using preference values for behaviours in a 1 km×1 km grid, i.e. we determined frequency of roaring and scent marking in each grid cell, and preference values for each grid cell. Grid preference was calculated as the ratio of use to availability [19], where a value of >1 indicated a preference and <1 indicated avoidance of an area. A two kilometre buffer zone along the boundary with the neighbouring pride was used to differentiate the response to social rather than resource stimuli. Resource limitation and preference was measured on one scale by contrasting observed ranges with available ranges, and also assessing the influence of rivers [29]. The latter was done by determining the preference for areas within 500 m of drainage lines or rivers.

Because all of the observations made in this study are based on a single male/coalition, a small female group, and a small subadult male group, we do not have a sample of truly independent samples and the conclusions should be viewed as preliminary, and will hopefully stimulate further work in this area.

## Results

### Description of ranging behaviour

**Figure 2:**
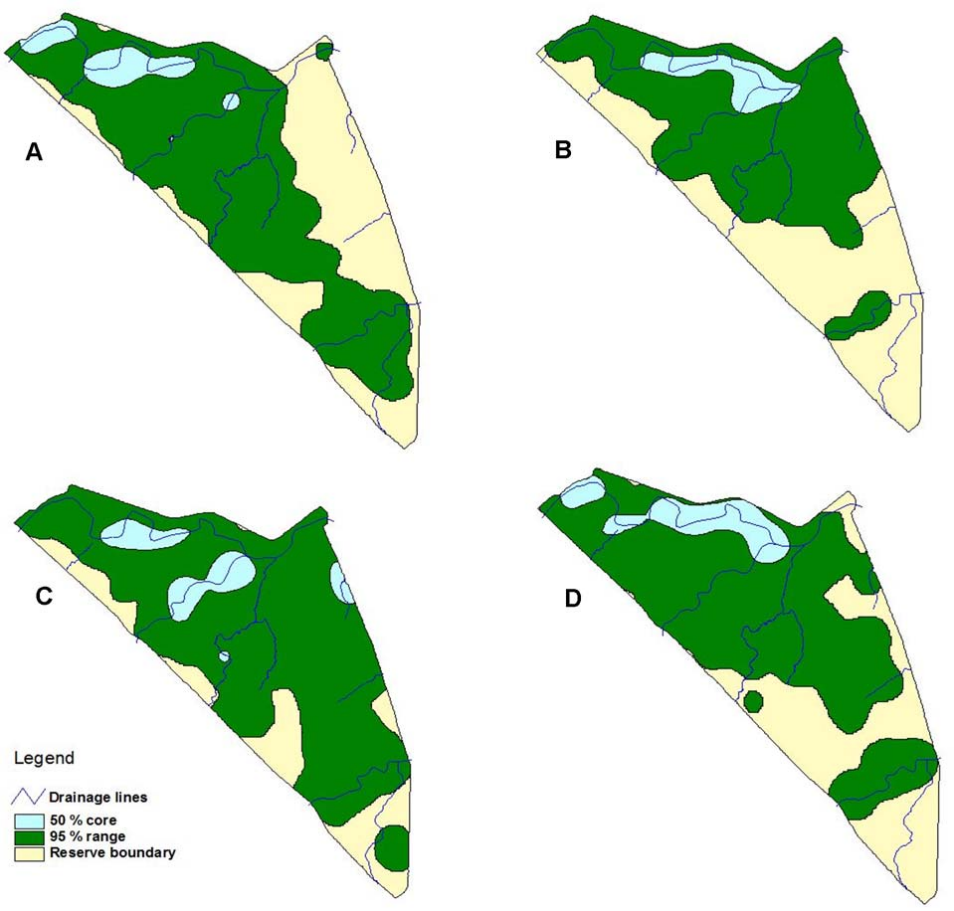
Home ranges of (A) male coalition, (B) single male, (C) females before male was removed, and (D) females after removal of the male.

Two male lions were released onto the reserve in September 1999, and two lionesses were released a month later. The range available to the lions was 80 km^2^. Table 1 shows the change in home range size during the study period. The pride's and the males' core home ranges were concentrated along the rivers (Fig. 2). The pride's home range (95% range 64.4 km^2^ and 50% core 10.3 km^2^) was larger than the males' home range (95% range 56.3 km^2^ and 50% core 5.0 km^2^) with the core double the size of that of the males. There was a noticeable difference between the summer home range (November – April) and the area utilised in winter (May – October). The lions (combined pride and males) utilised almost the whole reserve in summer (95% range 77.4 km^2^ and 50% core 10.6 km^2^) with a large core encompassing a larger area away from the rivers. The winter home range had a 95% range of 58.9 km^2^ and a 50% core of 6.1 km^2^. Almost 70% of the reserve lies within 500 m of generally permanent water, and 58.2% of the 95% summer home range, and 86.8% of the 50% summer core within 500 m of water; while 99.1% of the winter core range was within 500 m of water.

**Table 1:** Combined male and female annual minimum convex polygon and kernel home range sizes, illustrating the expansion of the home range from 2000 to 2005.

Home Range	95% range (km^2^)	50% core (km^2^)	Minimum convex polygon (km^2^) *
2000	35.0	1.2	76.9
2001	59.5	5.7	78.1
2002	63.5	7.2	74.7
2003	52.4	4.3	72.9
2004	59.7	5.5	73.6
2005	65.4	5.5	77.9
**Management action (splitting male coalition)**			
Before	68.8	6.3	78.6
After	66.0	5.1	78.1

* Area of clipped minimum convex polygon that falls within the reserve boundary.

The pride's home range expanded from 53.4 km^2^ to 56.8 km^2^ (Fig. 2). Before the male was removed the lionesses were observed to spend little or no time in the eastern side of the reserve, but included that area thereafter (Fig. 2). The pride males had a home range of 66.6 km^2^ and a core of 6.2 km^2^ that was reduced to 47.4 km^2^ with a core of 4.8 km^2^ when the male was removed (Fig. 2).

### Home range response to resource and social factors

Despite using an average of 82.2% (69.9–86.6%; n = 4) of the grid cells across the reserve, all lion groups selectively preferred only 21.7–31.3% (n = 4) of these. Almost all the cells in the eastern buffer zone were used (88.9–100%, n = 4) by the four lion groups (Table 2).

**Table 2:** The percentage of the reserve that is preferred, avoided, or used at availability by the different lion groups measured (a) across the whole reserve to indicate the proportion of the reserve that is utilised, and (b) within the 2 km buffer zone along the eastern boundary to indicate whether the buffer zone is preferred or avoided.

Preference (use/availability)	Male coalition (%)	Single male (%)	Females with coalition (%)	Females with single male (%)
**Over the whole reserve**				
Preference (>1)	27.7	21.7	26.5	31.3
At Availability (1)	13.3	12.0	10.8	8.4
Avoidance (<1)	59.0	66.3	62.7	60.3
**Within the 2 km buffer zone along eastern boundary**				
Preference (>1)	38.9	50.0	5.6	5.6
At Availability (1)	16.7	11.1	0	22.2
Avoidance (<1)	44.4	38.9	94.4	72.2

**Figure 3:**
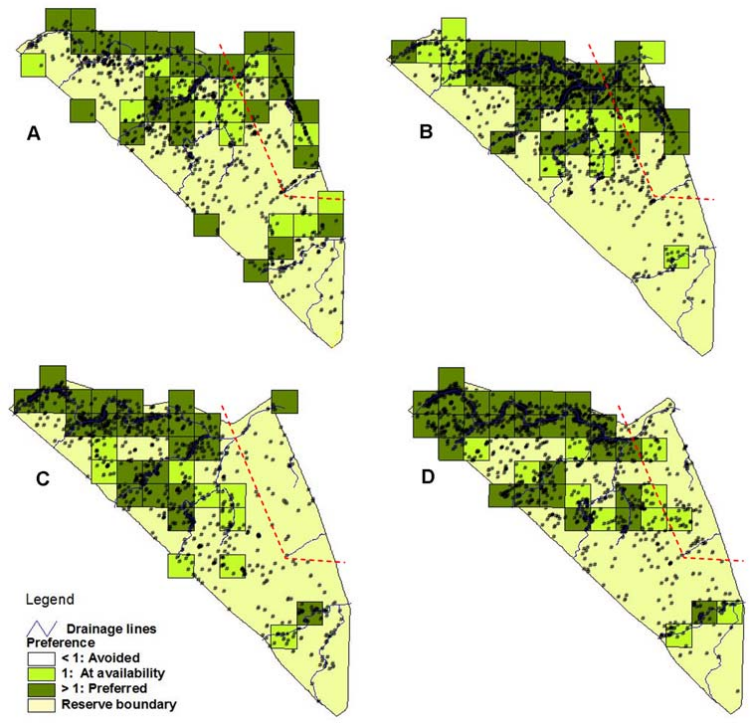
Spatial distribution of lions. Areas preferred, avoided, or used according to availability for (A) male coalition, (B) single male, (C) females before male was removed, and (D) females after removal of the male. The red line indicates the edge of the 2 km buffer zone from the adjacent lion population to the east.

The coalition preferred cells scattered around the reserve and spent more time in those areas than the single male did (Fig. 3). The single male preferred areas mostly in the northern half of the reserve, which coincided with the female's preference, and was also along the major drainage lines.

The areas within 500 m of drainage lines were most preferred by all lion groups with the females showing the highest preference for these areas.

### Territorial behaviour

**Figure 4:**
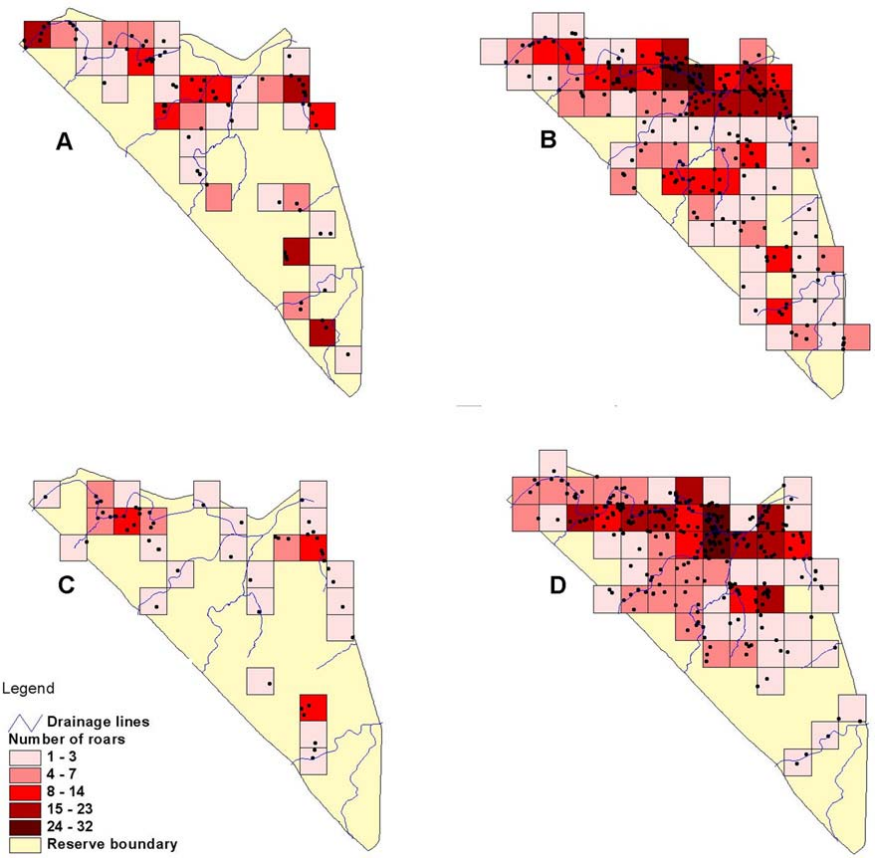
Spatial patterns in territorial behaviour indicated by density of behaviours across the reserve. Locations of (A) scent marks made by the pride males from 1999 to 2002, (B) scent marks by the single male from 2002–2005, (C) roars by the pride males from 1999 to 2002 and (D) roars by the single male from 2002–2005.

Both the coalition and single male showed stronger preference for the buffer area (coalition preferred 38.9% of buffer and only 27.7% of the rest of the reserve; and the single male preferred 50% of the buffer and only 21.7% of the rest of the reserve). Figure 2 indicates that the core ranges were predominantly along drainage lines and indicates preference for those areas, as can also be seen in Figure 3. Point distribution in Figure 3 indicates that both the coalition and single male concentrated on the core areas along the rivers, as well as along the eastern fenceline. Figure 4 indicates a similar pattern for locations where the males displayed territorial behaviour. The males therefore showed the most notable territorial behaviour in response to the social factor in the east, as well as resource factors by defending their prime habitat within their home range.

The coalition preferred, while the single male showed a slightly weaker preference for scent marking within the buffer zone along the eastern boundary. In terms of roaring, both the coalition and the single male strongly preferred using the buffer zone.

Overall 624 scent marks were observed by the pride male/s, as compared with only 119 excretions. Additionally the pride male/s were heard roaring 578 times (Table 3), and 74.3% (*n* = 1313) of all territorial scent marks and roars occurred within drainage lines or within 500 m of water.

**Table 3:** The effect of coalition size manipulation (male removal) on territorial behaviour of pride males.

Observation period	Days	Scent marks	Roars	Urinate / defecate
**Before male removal**				
Male	988	126	55	33
Female	988	2	24	20
	**Total**	**128**	**79**	**53**
**After male removal**				
Male	1130	328	352	58
Female	1130	0	80	28
Subadults	1130	1	14	8
	**Total**	**329**	**446**	**94**
**Nocturnal observation after male removal**				
Male	48	170	171	28
Female	48	0	62	18
Subadults	48	0	3	1
	**Total**	**170**	**236**	**47**

The single pride male scent marked at a rate of 1.1 scent marks/km. These included spay urinating on bushes (63.2%), of which he rubbed his body or head on the bush before urinating on 34% of the occasions, and urinating on the ground while scraping with the back feet (36.8%).

The single pride male roared at a rate of 0.6 roars/hour (*n* = 48). The male roared more frequently while alone than with pride members, with an average of 2.0 roars/hour while alone compared to 0.4 roars/hour when not alone.

**Figure 5:**
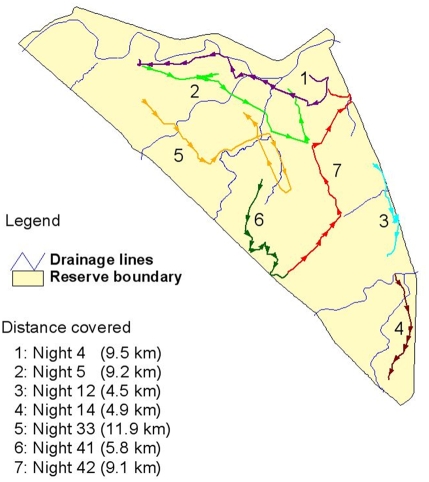
Selected nights showing movement paths travelled by the pride male, illustrating distance and path walked during territorial patrols.

The pride male covered an average distance of 0.45±0.07 km/hr (*n* = 48). Figure 5 indicates movement paths on selected nights where more than 4 km were covered. The largest distance covered in one six hour observation was 12.0 km. On nine nights the pride male and any associated lions with him did not walk at all. The lions had a kill on three of those and the male was mating on two of the others. The pride male seemed to cover more distance while on his own than when other members of the pride were with him. This could be a general pattern but equally the small sample size (*n* = 1) could probably be as good an explanation.

The distances he moved did not seem to be affected by cloud cover, time of night (17:00–00:00; 21:00–04:00; or 23:00–06:00), or the phase of the moon. However, there seemed to be an interaction between time of night and the moon phase, with the furthest distance being walked on full moon between 21:00–04:00 (mean 6.4 km, *n* = 4), and the shortest distances being walked on new moon between 17:00–23:00 (mean 0.3 km, *n* = 4). The male walked less than expected between 23:00–06:00 during all four moon phases, and more than expected between 21:00–04:00 during both full moon and the first quarter.

## Discussion

Lion home range sizes vary considerably across study areas, ranging from 20–45 km^2^ in places like Manyara National Park and Ngorongoro Crater [30], [31] to 2075 km^2^ in arid ecosystems such as Etosha National Park [32]. Even a study on the neighbouring reserve, Makalali, showed variability with home ranges varying from 24.9 km^2^ to 106.8 km^2^ [33]. Individual variability has been shown to be the largest source of variance in mean estimates of home ranges [34]. On the central and south-eastern basalt regions of Kruger National Park the home ranges were about 100 km^2^ [17], [35], whereas they were about 250 km^2^ on the northern basalt plains [36]. Thus in both Karongwe and Makalali lions seemed to occupy substantially smaller home ranges than in Kruger National Park. This was not due to the fence surrounding the reserve as the lions' overall range sizes were smaller than the reserve potentially allowed.

It has been variously suggested that home range size and configuration of large carnivores is influenced by patterns of resource distribution [7], [37], and by social effects [19]. In this study both the pride males were unaffected by the pride to the east using the buffer area according to its availability, i.e. the males seemed to use the resources of their territory regardless of social influence. The females, however, tended to avoid the buffer area seemingly displaying a negative response to the social influences from the east. Females and their cubs are violently affected by incoming males [38] and could be avoiding the area to prevent contact with potentially infanticidal males. [39] showed that new prides often settle adjacent to their natal range but the pride females on Karongwe, which originated from neighbouring Makalali, were nevertheless cautious of this boundary area. It should be remembered that the study is only based on one pride resulting in a small sample size which could affect the results.

The males and females showed strong preference for drainage lines and rivers. This could be largely due to shifts in prey availability, opportunities for prey capture [29], or cover and protection for cubs [40], particularly as the area was most strongly preferred by the lionesses. Karongwe and Makalali are both small reserves where prey movements are constrained by fences, the only local movements being that prey moves closer to riverine and other water rich areas in winter. It is also important to note that Karongwe was stocked above herbivore carrying capacity. Therefore, there was no resource limitation but the lions still tracked the prey across seasons, indicating that this could be a resource driver of spatial ecology.

Although the pair of males used the buffer zone according to its availability, both the coalition and single pride male showed preference for both scent marking and roaring in this area, reflecting the social influence from the east. The pride male did most territorial patrolling while alone, when he could cover more distance, and not place pride members at unnecessary risk in the event of an encounter. Lionesses were observed to scent mark and roar, but usually while in the presence of the pride male, probably because roaring increases their risk of attracting potentially infanticidal males [38]. [17] also noted that a lot of territorial behaviour occurred along drainage lines in the nearby Kruger National Park. This could be because rivers are often natural borders between home ranges [17], or are areas that offer male lions better hunting opportunities [41].

### Conclusions

Key results from our study are that male ranging behaviour is possibly not affected by coalition size, and suggests it is more driven by resources than social factors. Female ranging on the other hand seemed to be driven by social factors above resources. Males changed their territorial behaviour rather than ranging pattern in response to social influences. Although based on a small sample size, these results may have important implications for conservation management of small lion populations. Social effects from surrounding reserves may lead to heterogeneity in ecological influences of lions, and need to be factored into management of prey populations (e.g. harvesting locations). Further, the removal of a male seems to have the advantage of decreasing prey off-take, but does not fundamentally shift ranging behaviour, even in the presence of a heterogeneous social influence. This is relevant to shared-ownership reserves that have traversing restrictions for various operators. By the same token, social effects may influence the spatial distribution of tourism-attractive behaviours such as roaring and scent marking. Understanding of fundamental drivers of territorial behaviour allows better planning and management of adaptively managed reserves, and these results can potentially contribute to the management of other territorial species.
